# The gene *fmt*, encoding tRNA^fMet^-formyl transferase, is essential for normal growth of *M*. *bovis*, but not for viability

**DOI:** 10.1038/s41598-017-15618-9

**Published:** 2017-11-09

**Authors:** Miriam Vanunu, Ziv Lang, Daniel Barkan

**Affiliations:** Koret School of Veterinary Medicine, The Robert H. Smith Faculty of Agriculture, Food and Environment, The Hebrew University of Jerusalem, Rehovot, Israel

## Abstract

*Mycobacterium tuberculosis* is a major health threat, necessitating novel drug targets. Protein synthesis in bacteria uses initiator tRNA_i_ charged with formylated methionine residue. Deletion of the formylase gene, tRNA^fMet^-formyl transferase (*fmt*), causes severe growth-retardation in *E*. *coli* and in *S*. *pneumoniae*, but not in *P*. *aeruginosa* or *S*. *aureus*. *fmt* was predicted to be essential in *M*. *tuberculosis* by transposon library analysis, but this was never formally tested in any mycobacteria. We performed a targeted deletion of *fmt* in *M*. *smegmatis* as well as Mtb-complex (*M*. *bovis*). In both cases, we created a mero-diploid strain, deleted the native gene by two-step allelic exchange or specialized-phage transduction, and then removed the complementing gene to create full deletion mutants. In *M*. *smegmatis* a full deletion strain could be easily created. In contrast, in *M*. *bovis*-BCG, a full deletion strain could only be created after incubation of 6 weeks, with a generation time ~2 times longer than for wt bacteria. Our results confirm the importance of this gene in pathogenic mycobacteria, but as the deletion mutant is viable, validity of *fmt* as a drug target remains unclear. Our results also refute the previous reports that *fmt* is essential in *M*. *tuberculosis*-complex.

## Introduction

*Mycobacterium tuberculosis* (Mtb) is a continuous world health problem by causing tuberculosis disease, and novel drugs to fight this malady are in dire need^1^. Identifying physiological and biochemical processes that are both unique to the bacterium (as opposed to mammals, and especially humans) and also essential to its viability is therefore important, as it may offer the discovery of novel drug targets. Prokaryotic protein synthesis differs in several steps from that of eukaryotes, therefore offering the potential of being such a unique process. Bacteria and higher eukaryotes use methionine as the initiator amino acid - however, in bacteria this methionine is a formylated one (fMet)^2^. The tRNA for formylated methionine (initiator tRNA, tRNA^fMet^_i_) differs from that of regular methionine (tRNA^Met^)^3^. Methionine can be loaded onto either of the tRNA molecules, but when loaded onto tRNA^fMet^_i_, it is formylated by Formyl-Methionione Transferase (FMT), encoded by the gene *fmt*. After the incorporation of fMet into the peptide chain, the formyl group is first removed by Peptide Deformylase (encoded by *def*)^4^, and then the methionine itself is cleaved off by Methionine Aminopeptidases (encoded in Mtb by *mapA* and *mapB*)^5^. Removal of this methionine is common to, and essential in all living cells^6^; However, the formylation and deformylation of the initiating methionine is unique to bacteria (and mitochondria). Despite this uniqueness, surprisingly little is published on the essentiality of this pathway to different bacterial species. The essentiality of *fmt* was suggested in *streptococcus pneumonia*, where *fmt* could not be genetically deleted^7^ (although the methodology of the deletion attempts was not clearly described). In *E*. *coli*, a targeted deletion resulted in severe growth retardation, reducing growth rate *circa* 12-fold^8^. However, a later study found the same deletion reduced the growth of *Pseudomonas aeruginosa* by only 3 fold^9^, and another study found only mild growth effect on *Staphylococcus aureus* ^10^. Another study found deletion of mitochondrial *fmt* in *Saccharomyces cerevisiae* results in a very mild phenotype of a longer lag on non-fermentable media^11^. We found no other studies addressing this issue in additional bacteria, and the essentiality in mycobacteria was never formally examined in a definitive way by gene deletions - although three independent transposon mutant analysis experiments and publications in *M*. *tuberculosis* ^12–14^ suggested the gene was indeed essential. However, gene essentiality predictions based on transposon mutant libraries may erroneously predict essentiality in genes where inactivation causes substantial, even if not complete, growth retardation.

Of note, gene essentiality in mycobacteria is sometimes checked by examining the extrapolating data generated in the model organism *Mycobacterium smegmatis*. However, *M*. *smegmatis* is a soil organism, with a genome of 6.5 million base pairs, whereas Mtb and *M*. *bovis* are intra-cellular pathogen, with a genome of 4.5 million bp. *M*. *smegmatis* therefore has the potential of possessing accessory metabolic pathways, and extrapolating essentiality data from it to Mtb may be misleading.

We therefore decided to genetically examine the essentiality of *fmt* – first in *M*. *smegmatis*, and then in Mtb-complex (*Mycobacterium bovis* BCG). For this purpose, we first pre-complemented the bacterium with an additional copy of the target gene (*Mtbfmt*, *Rv1406*) at the *attb* site, performed a targeted deletion of the native gene, and then proceeded to removing the complementing copy from the *attb* site using the highly efficient “*attb*-cassette exchange” technique^15^. Because the cassette exchange is a very efficient procedure that can take place in thousands of bacteria, failure to perform this exchange is highly suggestive of absolute essentiality, whereas relative essentiality (severe growth disadvantage) will result in reduced colony number or longer time-to-colony, but not an absolute absence of colonies.

## Results

### Testing the essentiality of *fmt* in *M*. *smegmatis*

**Figure 1:**
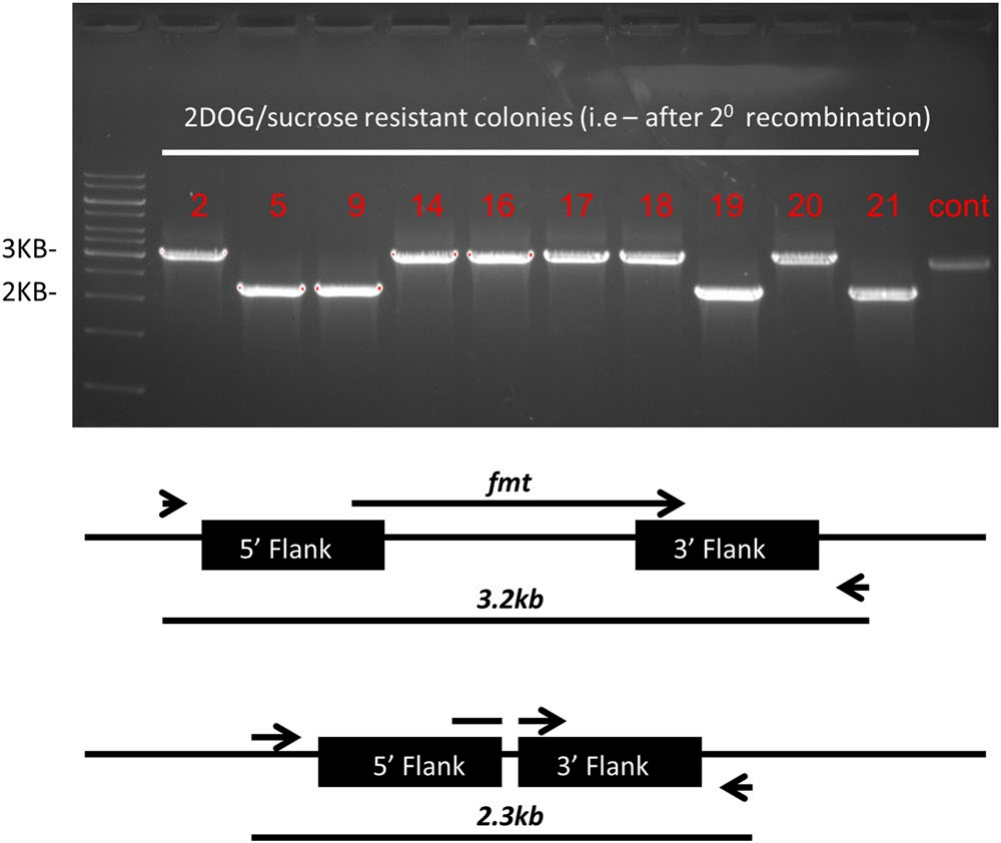
Creation and confirmation of an *fmt* deletion mutant in *M*. *smegmatis*. 10 of the colonies that grew on 2DOG/sucrose were analyzed by PCR (primers fmtdelchq1,2). Wt genotype results in a 3.2 kb product (control, rightmost lane), whereas *Δfmt* results in 2.3 kb. See illustration below. The primers are external to the flanking regions used for recombination. Clone 5 (*) was chosen for continuation, and called mDB21.

To do this, we first created a mero-diploid mutant of *M*. *smegmatis*, by inserting an additional copy of *fmt* into the *attb* site, under kanamycin selection. We used the Mtb*fmt* gene (*Rv1406*), and placed it under anhydrotetracycline (AHT) controlled promoter, in a manner that addition of AHT allows expression, whereas with no AHT the expression is minimal, if at all^16^. The plasmid containing the AHT-*fmt*, integrating at the *attb* site, was called pDB117, and was inserted into wt *M*. *smegmatis* (MC^2^-155) by electroporation. We then used the two-step allelic exchange technique to delete the native *fmt* gene (Msmeg_3064)^17^, using the plasmid pDB178. After the first crossover event we allowed the bacteria to divide for circa 15 generation times (in media supplemented with AHT), and then plated on plates with the counterselection agents sucrose and 2-deoxy-galactose (as well as AHT), selecting for a second recombination event that can lead to either reversal to wt genotype, or to a deletion genotype^18^. 25 colonies were picked, grown in subculture, and those that grew were analyzed by PCR for identification of *fmt* deletions mutants (Fig. 1). As seen, several *fmt* deletion mutants were obtained, one of them was arbitrarily chosen for the continuation of research, and called mDB21 (*Δfmt*, *attb:kana:Mtbfmt-AHT*).

To test if *fmt* was essential, we first tested if mDB21 growth was dependent on AHT, controlling *fmt* expression. We therefore grew mDB21 with AHT, washed it twice by centrifugation in media to remove all residual AHT, and both inoculated it into media with or without AHT, and plated on 7H10 plates with or without AHT. Surprisingly, the bacteria grew well both in the presence and absence of AHT (Supp. Figure 1). We passed the culture several times into fresh media to ensure all AHT in the media, as well as in the bacteria, was depleted – however, the bacteria continued to grow in apparently normal rate. This could suggest that *fmt* was not essential, that an escape mutation occurred at the AHT controlled promotor, or that the very low (but existing) expression of *fmt* in the absence of AHT is sufficient to support growth.

**Figure 2:**
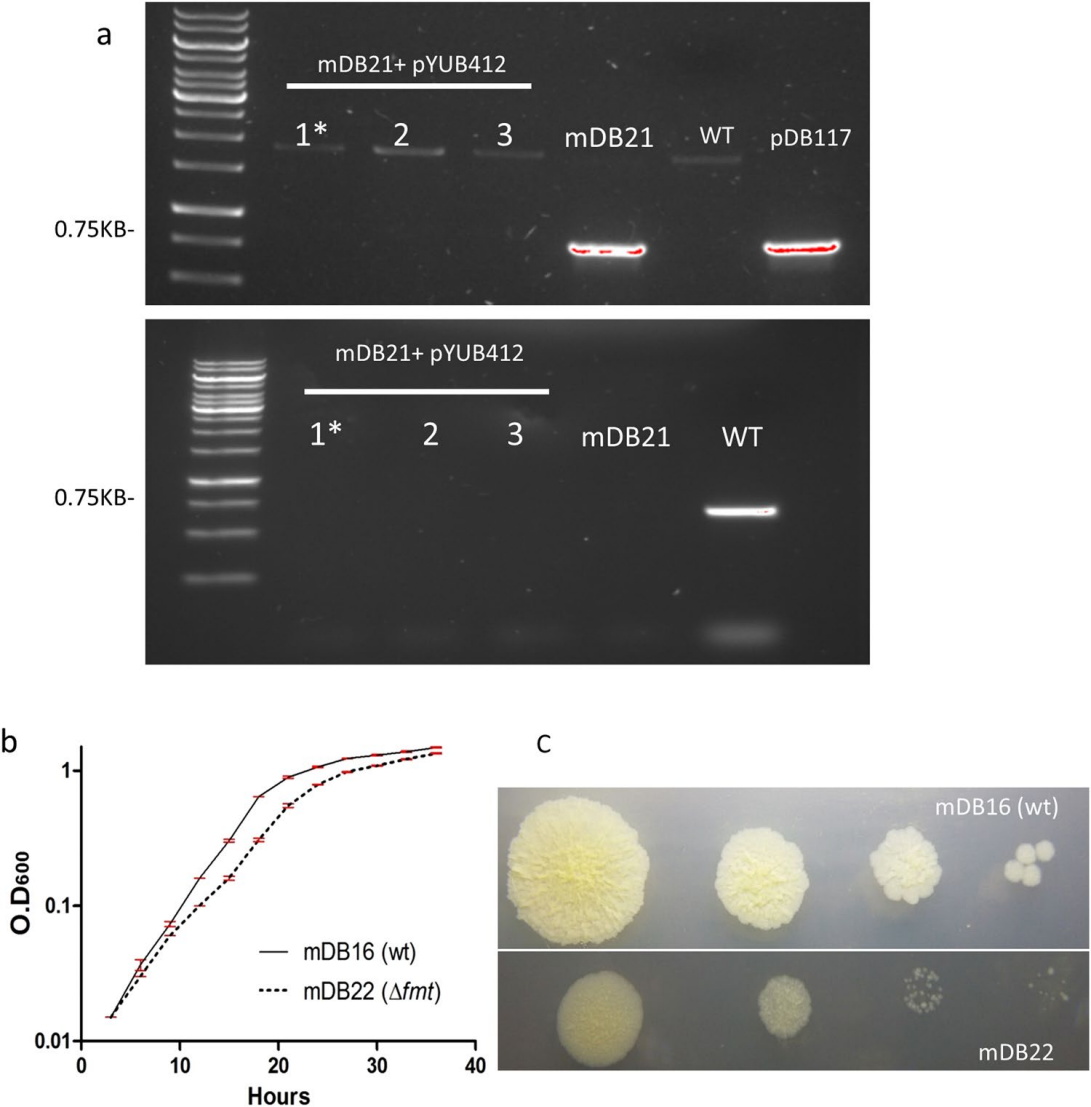
mDB22, a complete deletion mutant of *fmt*, has a growth defect compared to wt-like *M*. *smegmatis*. (**a**) After electroporation with pYUB412, mDB21+pYUB412 has neither *Mtbfm*t (top panel) nor *M_Smgfmt* (bottom panel). For *Mtbfmt* amplification, we used primers fmtTbup-do (expected product 0.75Kb), and for *M_Smgfmt*, primers fmtSmgup-do (0.6Kb). As seen on *M*. *smegmatis* and mDB117, the primers do not cross-react. Colony 1(*) of mDB21+pYUB412 was named mDB22, and used for further experiments. (**b**) Growth of mDB22 (full deletion mutant) as compared to wt-like *M*. *smegmatis* (*attb:hyg*, mDB16), at 37 °C. mDB22 has a small but consistent growth defect. Error bars are Standard Error of the Mean (SEM). (**c**) Dilution spot-plating of mDB22 (bottom) versus mDB16 (top). Despite slightly higher numbers of mDB22, the growth defect is easily seen.

To test which of these possibilities is correct, we decided to completely remove the complementing Mtb*fmt* gene from the genome. To do this, we electroporated the *attb*-integrating, hygromycin-selected plasmid pYUB412, with no *fmt* on it. A successful electroporation will result in a complete null mutant^17^, and therefore if the gene is essential, no colonies will appear. If the gene is essential but a compensatory (‘escape’) mutation occurred, handful of colonies may appear (as a compensatory mutation is a rare event). However, if the gene is not essential, the cassette exchange will be highly efficient, and many colonies will appear. Indeed, electroporation of pYUB412 resulted in hundreds of colonies. Three arbitrary colonies were picked, and shown by PCR to be complete deletion mutants of *fmt* (Fig. 2a), with neither *Msmgfmt* nor *Mtbfmt*. One of these colonies was arbitrarily chosen for continuation of research, and named mDB22 (*Δfmt*, *attb:hyg*).

To see if deletion of *fmt* had an effect on growth rate, we followed the O.D_600_ of mDB22 as compared with wt *M*. *smegmatis* (with pYUB412, to prevent bias from the presence of an aminoglycoside resistance gene) for over 24 hours. mDB22 had a trivial but consistent growth disadvantage in liquid media (Fig. 2b), that was more pronounced in a longer lag-period. Growth during the logarithmic-phase was almost identical to that of wt (the linear/linear growth curves are presented in Supp. Figure 2). The growth retardation was also very evident on solid agar plates – as shown by serial-dilution plating of mDB22 and wt *M*. *smegmatis* (Fig. 2c), although it is impossible to say if this is due to slower growth, or longer lag-period. Altogether, it appears *fmt* was not essential for near-normal *M*. *smegmatis* in 7H9 culture.

### Testing the essentiality of *fmt* in the *M*. *tuberculosis*-complex bacterium *M*. *bovis* BCG

**Figure 3:**
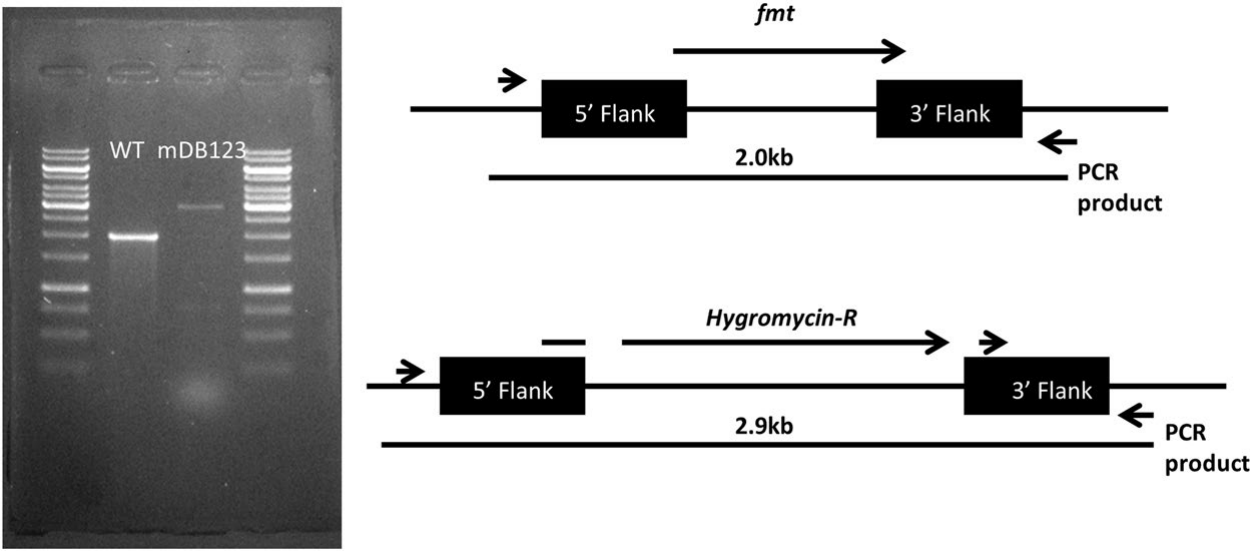
Creation and confirmation of a deletion mutant of *fmt* in BCG russia. BCG wt and a hygromycin-resistant mutant were analyzed by PCR using primers fmtKOchqD,U. The primers produce a 2 Kb product in wt, and 2.9 Kb in a correct deletion mutant (see illustration on the right). The correct mutant was called mDB123.

**Figure 4:**
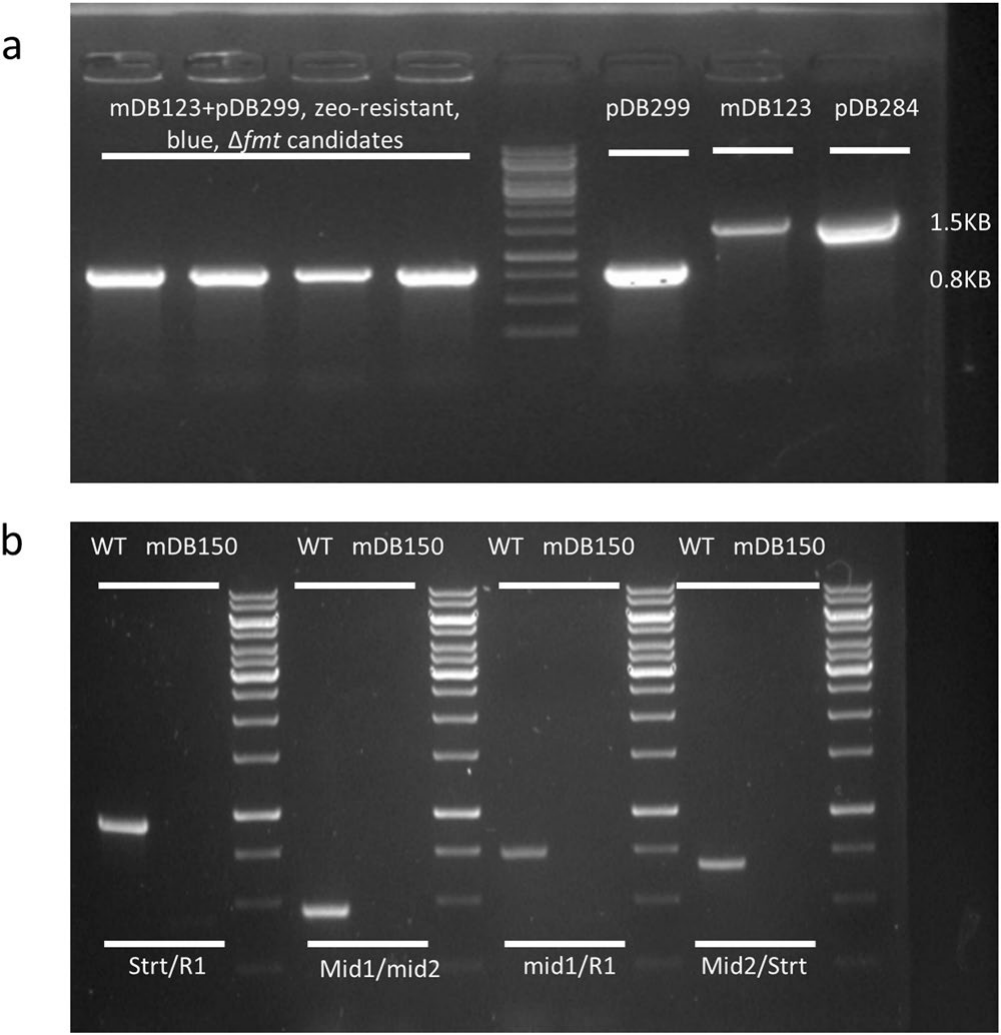
The kanamycin-selected cassette pDB284 could be exchanged for the zeocin-selected pDB299, that has no *fmt*, thus producing a full deletion mutant. (**a**) mDB123 was electroporated by pDB299 and plated on zeocin. Blue colonies were picked and analyzed by PCR using primers *intEnd*, *OriEstart*. The primers bind to pDB284 (and thus, the “parent strain” mDB123), producing a 1532 bp product, and to pDB299 (and to strains where a correct exchange occurred), producing a 830 bp product. All 4 blue colonies were correct transformants, and one of them (*) was chosen for continuation, and named mDB150 (Δ*fmt:hyg; attb:zeo:lacZ*). (**b**) Four different PCR reaction [using primers *strt/R1* (950 bp), *mid1/mid2* (480 bp), *mid1/R1* (750 bp), *mid2/strt* (670 bp)], all amplifying *Mtbfmt*, were performed on wt and mDB150. All reactions failed to detect presence of *fmt* in mDB150.

**Figure 5:**
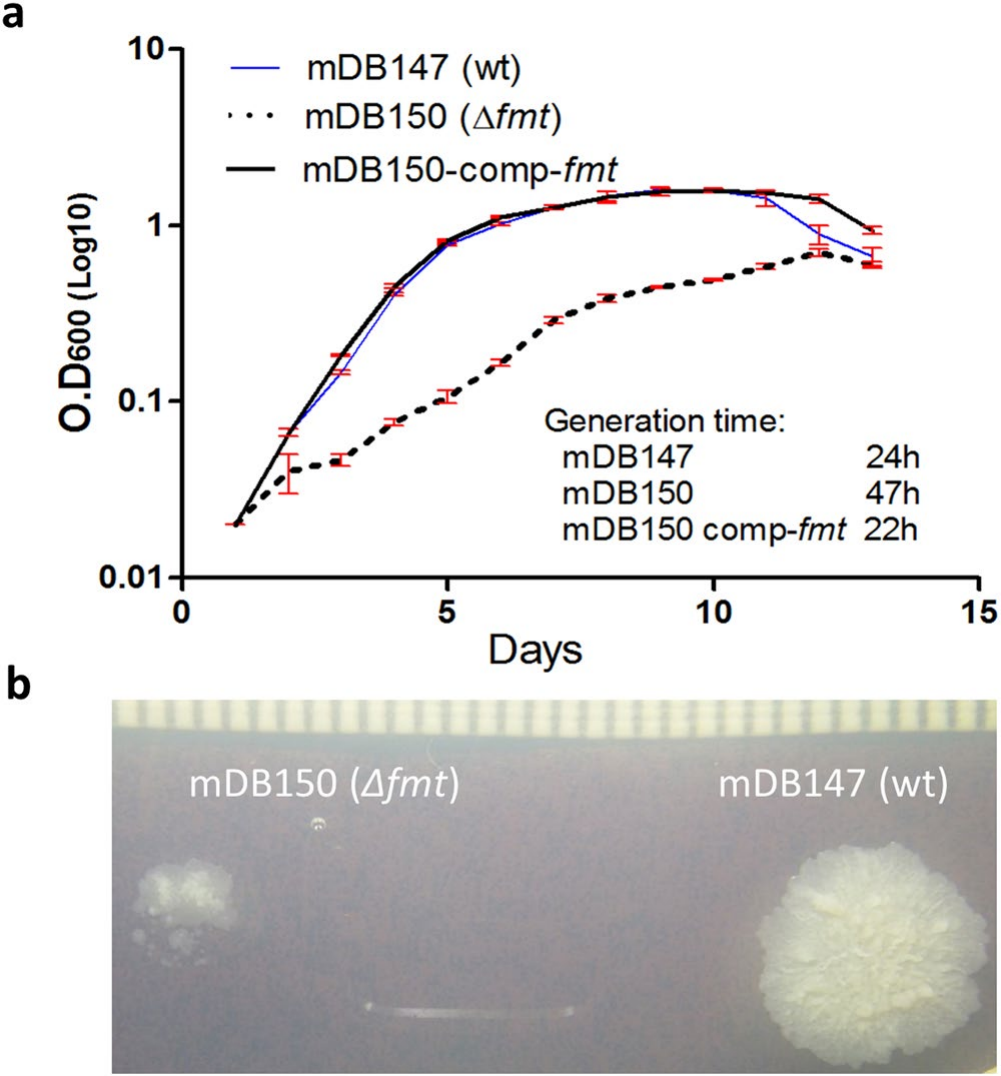
BCG *Δfmt* mutant (mDB150) has a significant growth defect as compared to the wt-like mutant (mDB147). (**a**) mDB150 (dotted black),mDB147 (thin blue) and mDB150-comp-*fmt* (thick black. Also called mDB168) were grown in triplicates, from an initial O.D_600_ of 0.015, and growth was followed daily. (As the triplicates grew almost identically, the error bars are almost flat in most time-points) (Error bars are Standard Error of the Mean, SEM). (**b**) 50 cfu of mDB150 and mDB147 were spotted on 7H10/hyg50/zeo33 plates. The picture was taken after 40 days incubation.

Considering *M*. *smegmatis*, as noted before, may have multiple metabolic pathways that do not exist in the obligatory pathogen Mtb, and therefore genes not essential in it may be essential in Mtb; and that eventual drug development will target Mtb and not *M*. *smegmatis*, we decided to test the essentiality of *fmt* in Mtb-complex bacteria *M*. *bovis*. We therefore created a mero-diploid mutant of BCG *subsp*. *russia*, with an additional copy of *Mtbfmt* (*Rv1406*) (plasmid pDB284) at the *attb* site (Mutant mDB113). This time we used the native *fmt* promotor and not the AHT controlled one, for simplicity reasons. We then proceeded to deletion of the native gene (*Mb1441*, identical to *Rv1406*) by the specialized transducing mycobacteriophage phDB30, replacing the gene with a hygromycin resistance marker^19^. A successful deletion was confirmed by PCR (Fig. 3), and the resulting mutant was called mDB123 (*Δfmt:hyg*, *attb:kana:fmt*). To examine if *fmt* was essential, we again opted for a marker exchange experiment: we electroporated mDB123 with either pDB290 (an *attp* integrating plasmid with zeocin selection, as well as a functional copy of *fmt*), or with the control plasmid pDB299 (*attb* integrating, zeocin resistance, *lacZ* positive, but no *fmt*). We expect to get multiple colonies with the pDB290 electroporation, as this will simply exchange the resistance cassette, with no effect on *fmt* genotype. When electroporating the empty pDB299, two possibilities exist: in case *fmt* is not essential, we expect to get multiple colonies that are zeocin resistant and kanamycin sensitive (and of blue color, when IPTG/Xgal is added to the media). In contrast, if *fmt* is essential, we expect to get no colonies at all. Indeed, when we electroporated mDB123 with either pDB290 or pDB299 (in similar amounts of DNA), after 3 week-time we got multiple colonies on the pDB290 plate, but no colonies on the pDB299 plate, leading us at first to believe that in Mtb-complex, in contrast to *M*. *smegmatis*, *fmt* gene is genetically essential. However, continued incubation of the agar plates eventually yielded, after over 6 weeks, multiple tiny blue colonies. Several of these colonies were picked, and after long recovery time, yielded turbid cultures. When checked by PCR, the cassette exchange of the kanamycin-selected, *fmt*-containing pDB284 for the zeocin-selected, *fmt*-lacking pDB299 was confirmed (Fig. 4a), and several PCR reactions with different primer pairs failed to detect *fmt* in the new mutant (Fig. 4b). Taken together with the blue color of the colony on Xgal, these findings confirmed the deletion of *fmt*. The mutant was named mDB150 (*Δfmt:hyg; attb:zeo:lacZ*), whereas its complemented counterpart was named mDB147 (*Δfmt:hyg; attb:zeo:fmt*). In sharp contrast with the near-normal growth phenotype in the *M*. *smegmatis Δfmt* mutant, the *Δfmt* mutant in BCG both took over 6 weeks on 7H10 plates to form colonies (although in large numbers), and was very slow to recover in liquid 7H9 media. We therefore formally tested growth curves of mDB150 versus the complemented mDB147. The deletion mutant had a substantial growth retardation phenotype, as seen in a long lag-period, only brief logarithmic phase, and a lower plateau as compared to wt bacteria (Fig. 5a) when grown in liquid, as well as spot-plating on solid agar (Fig. 5b). The calculated generation time (doubling-time) for mDB150 was twice as long as for mDB147 (approximately 47 h Vs. 24 h for wt). The linear/linear growth curve is presented in Supp. Figure 2. The slow growth phenotype was reversed by complementation of *fmt* on an episomal plasmid (mutant mDB168, also called mDB150-comp-*fmt*) (Fig. 5a).

### Total proteomic analysis of *Δfmt* mutant Vs. wt

Our result, showing a 2-fold increase in generation time in the *Δfmt* (mDB150) mutant as compared to wt (mDB147) is in agreement with previously described effects in some bactria, but is milder than in most. To further characterize the cellular and metabolic effect of *fmt* deletion, we performed a total, unbiased proteomics analysis of mDB147 Vs. mDB150. The results are presented in Supp. 2 in the form of an Excel spread-sheet. When the threshold for change was +/− two-fold difference, and the P value threshold was set at 0.05, 98 proteins (in addition to FMT itself) were found to be downregulated in the mutant (mDB150) as compared to wt (mDB147). Other 52 proteins were upregulated in mDB150 as compared with wt. However, in most proteins the change was mild, and considering the total number of proteins was 2412, a P value of 0.05 was probably too permissive. When we re-analyzed the data with more stringent parameters of +/− 5-fold and a P value smaller than 0.01, only 10 proteins were significantly changed in mDB150 compared to wt - 7 proteins downregulated, and 3 upregulated. A search of the literature on what is known of these proteins/genes did not come up any obvious common denominator or explanation as to the change in their levels.

## Discussion

Identification of metabolic processes unique to bacteria in general, and the specific bacteria against which drugs are developed, is an important step in drug-target identification. Here we show that the gene *fmt*, encoding the enzyme tRNA formyl-methionine transferase, is not essential in the soil organism *M*. *smegmatis* (often used as a model for mycobacterial physiology), but its deletion causes substantial growth retardation in the Mtb-complex pathogen *Mycobacterium bovis*. These results are in contrast to the results obtained from a transposon-mutant library analysis, which listed *fm*t among essential genes in Mtb^11^. The substantial growth delay of this mutant (time-to-colony of 6 weeks), would easily cause the viability of the deletion mutant to be missed, when it is in competition with thousands of “healthy” clones, as it happens in transposon-mutant libraries. This should lead to caution when interpreting this kind of analysis for other genes as well.

*fmt* was previously suggested to be essential to the viability of *S*. *pneumonia*e^7^ (although the methodology was not clearly described), and to normal growth in *E*. *coli*, as a deletion mutant grew 12 fold slower than wt^8^. At first this was thought to be representative of most bacteria – or at least those related to enterobacteriaceae. However, a later study found that a similar deletion in the related organism *P*. *aeruginosa* had much milder effect on growth, with the mutant growing only 3 times slower than wt^9^. A deletion mutant in *S*. *aureus* was also viable, although growth was mildly compromised, and susceptibility to some antibiotics was enhances. Most of these bacteria simply manage to incorporate non-formylated methionine as the initiating amino acid^9,20^. It appears that a metabolic adaptation, rather than an additional genetic change, enables the survival of the deletion mutants. A study done in *Salmonella enterica* showed increasing levels of tRNA^fMet^_i_ (initiator tRNA) can compensate *fmt* inactivity^21^. Our results in *M*. *smegmatis* show *fmt* is either non-essential in this organism, with only mild effect of bacterial growth (at least in normal culture conditions). An alternative explanation could be that in those colonies we recovered, a compensating mutation arose, allowing for growth of the *Δfmt* mutant. However, the fact we simultaneously got hundreds of *fmt* deletion mutants makes the possibility of a ‘compensating mutation’ highly unlikely. In *M*. *smegmatis*, the effect of the deletion appears to be the mildest of all tested bacteria – either because of higher ability to use non-formylated methionine, high expression of tRNA_i_, or a putative existence of other accessory metabolic pathways.

The results we got in *M*. *bovis* were much more striking: as in the case of *M*. *smegmatis*, we got multiple colonies of complete *fmt* deletion mutants (easiely identified and separated from background colonies by the *lacZ* gene on the control plasmid) – thus strongly suggesting against a compensating mutation. However, the effect on growth was much more prominent, with up to 6–7 weeks required for colony formation of the mutant, slow growth in liquid media, and a measured doubling time of ~48 hours. This suggests that *fmt* (and methionine formylation) is much more important in *M*. *bovis* (and Mtb) than in *M*. *smegmatis*, and that resistance to the deletion effect is less robust. Still, the effect on viability and growth rate was less pronounced than in some other bacteria (like the 12 fold growth reduction in *E*. *coli*), and more closely resembled the milder effect in *P*. *aeruginosa* or *S*. *aureus*. Based on this two-three fold growth retardation, the value of *fmt* targeting in tuberculosis treatment remains unclear. Targeting this pathway could potentially augment other treatments, especially those involved in protein synthesis (like amikacin, streptomycin, linezolid and maybe others). However, this question remains open for future research. Also of note is that *fmt* is present in human mitochondria, and mutations in the mitochondrial gene were connected to rare human genetic diseases^22^. This further complicates a potential role for *fmt* as a viable drug target, unless high specificity can be achieved.

Following the incorporation of the formylated methionine as the initiator amino acid, the formyl group is removed by Peptide deformylase (encoded by *def*). *def* essentiality was not examined in *M*. *smegmatis*, but in *M*. *bovis* it was shown to be essential^23^. In other bacteria, *def* was also shown to be essential, but resistant mutants arose frequently – many of them by inactivating mutations in the *fmt* gene, making the activity of *def* unnecessary^21,24^. This is one example of how simple compensatory mutations can make an essential gene become non-essential, with unpredictable effect on pathogenesis. It remains unclear though, if a compensatory mutation in *def* will enable Mtb normal growth when *fmt* is inhibited, for the mechanism behind each essentiality and lethality is different.

The whole issue of “gene essentiality” raises some philosophical questions of the actual meaning of “essential genes”. In *E*. *coli*, *fmt* was called essential even though deletion mutants could be obtained – because growth was 12 times slower in the mutant. In *P*. *aeruginosa*, growth was only 3 times slower, which was enough to call the gene “non-essential”, and in *S*. *aureus* growth impairment was even milder. Our experiments in *M*. *bovis* suggest *fmt* is essential for normal growth in Mtb, but the clinical meaning of this is unclear - it appears likely that Mtb bacteria growing 10 times slower than wild-type will have a much lower pathogenic potential. However, bacteria that grow 2–3 times slower, if not impaired in any way in intracellular survival, may theoretically still cause disease. Clinical experience shows that Mtb bacteria isolated from tuberculosis patients grow in “normal” rate, but very slow phenotypes may have been missed and dubbed “culture negative, PCR positive” cases.

Of note, our results also show the limitations of two widely used systems - using *M*. *smegmatis* as a model for pathogenic mycobacteria, and the use of transposon-mutant library for gene essentiality testing. *M*. *smegmatis* is not a pathogen, and therefore is rarely used in any pathogenesis studies (where its role is mostly limited to being a negative control). However, it is widely used for physiological studies such as DNA synthesis and repair, stress and starvation responses, as well as other biochemical processes. In many cases *M*. *smegmatis* is an excellent model – however, our results show that data obtained in it should be corroborated in the actual target bacteria – in this case, Mtb-complex. This is also true regarding essentiality data from transposon-mutant libraries – actual essentiality can probably only be predicted by targeted deletion experiment, and library results can only serve as preliminary guides.

## Materials and Methods

### Bacteria and growth conditions

*Mycobacterium smegmatis* and BCG (subsp. *russia*) were grown in 7H9 liquid media and on 7H10 plates supplemented by glycerol, Albumin-Dextrose-NaCl (ADS) and tween 80 (the latter in 7H9 only), in previously published and widely used concentration. The concentration of antibiotics was 20 µg/ml for kanamycin, 50 µg/ml for hygromycin, 20 µg/ml for streptomycin and 25 µg/ml for zeocin. AnHydroTetracycline (AHT) was added at 50 ng/ml where needed, and the negative selection agents were 5% sucrose and 0.2% 2-deoxy-galactose. Electroporation was done as previously widely described.

### *fmt* (Msmeg_3064) deletion in *M*. *smegmatis*

deletion was done in the two-step allelic exchange method, using the plasmid pDB178 that carries the upstream and downstream 600 bp flanking regions of the *fmt* gene. The plasmid also carries resistance to zeocin, streptomycin, as well as the negative selection genes *galK* and *sacB*, and the fluorophore *mCherry*, to assist in transformant selection and counter selection. Bacteria were first electroporated and plated on zeocin and streptomycin, pink colonies were chosen and were checked by PCR to have undergone one recombination event. The bacteria were then grown without antibiotics for 15 generations, and plated on 0.2% 2DOG and 5% sucrose. Colonies were picked and analyzed by PCR, with primers outside of the target gene (and external to the flanking region). A successful deletion results in a PCR product of 2.3 kb, as compared to 3.2 kb in wt.

### Expressing *fmt* from an AHT sensitive promotor

Mtb *fmt* was PCR amplified from Mtb genomic DNA, and cloned under the AHT sensitive promotor previously described, to create the plasmid pDB117. To allow expression from the promotor, 50 ng/ml of AHT were added to liquid or solid media.

### Deletion of *fmt* (*Rv1406*, *Mb1441*) in BCG

the deletion was done using a temperature-sensitive, specialized transducing mycobacteriophage based on phAE87, as previously widely described^25^. Briefly, the flanking regions of *fmt* were cloned on either side of the hygromycin resistance gene on a pMSG360, creating pDB245. This plasmid was used to create the phage phDB30 by recombineering in EL350 bacteria that carry the phagemid of phAE87. The phage was used to infect BCG at 39 C for 6 hours, bacteria were then plated on 7H10 plates with hygromycin, and colonies picked after 3–4 weeks. Colonies were analyzed by PCR with primers outside of the flanking regions (Fig. 3). A successful replacement of the *fmt* gene by *hyg-R* results in a PCR product of 2.9 kb, as opposed to 2.0 kb in wt.

### Cassette exchange

exchange of an attb-integrated plasmid for another one was previously described^17^. Briefly, the new plasmid (with a resistance marker different from the marker of the plasmid presently integrated) is electroporated as per protocol, and the bacteria are plated on 7H10 plates with the new antibiotic. If the plasmid can be exchanged (that is, if removal of the existing plasmid does not cause a functional deletion of an essential gene, or the incoming plasmid has the same gene), colonies will appear. If not, no colonies, or only background ones, will appear. Here, we used this technique to create a full *fmt* deletion mutant in *M*. *smegmatis* (by electroporating pYUB412 into mDB21), and similarly to create full *fmt* KO mutant in BCG (by introducing pDB299 into mDB123).

### Complementation

to complement mDB150 with a functional *fmt* gene, we prepared the plasmid pDB332, by cloning Mtb*fmt* into the pDB151 episomal plasmid, conferring resistance to kanamycin, and also expressing the red fluorophore *mCherry*, for ease of selection. pDB332 was electroporated into mDB150, and red colonies were picked after 3–4 weeks (as the slow growth phenotype reversed to normal growth).

### Proteomic analysis

mDB150 and mDB147 were grown (in triplicates) to an OD of 0.3 in a 15 ml volume, washed in PBS, and sent for proteomic analysis (The Smoler Protein Research Center, the Technion, Haifa). Proteins were extracted, digested by trypsin, analyzed by LC-MS/MS on Q exactive (Thermo) and identified by Maxquant software version 1.5.2.8 Vs. the *Mycobacterium tuberculosis* H37Rv databases and against decoy databases (in order to determine the false discovery rate). Statistical analysis was done using the Perseus software (Mathias Mann, Max Planck Institute).

## Electronic supplementary material

Supplementary data

Supplementary dataset 1
