# Supplementary material for: The gene fmt, encoding tRNAfMet-formyl transferase, is essential for normal growth of M. bovis, but not for viability
Source: Sci Rep. 2017 Nov 9;7:15161. doi: 10.1038/s41598-017-15618-9 (PMC5680289; doi:10.1038/s41598-017-15618-9)
Supplement: Supplementary file 1 — Supplementary data [file 41598_2017_15618_MOESM1_ESM.pdf]

Title:

The gene *fmt*, encoding tRNA^{fMet}-formyl transferase, is essential for normal growth of *M. bovis*, but not for viability.

Miriam Vanunu[1§], Ziv Lang[1§], Daniel Barkan[1,*]

1. Koret School of Veterinary Medicine, The Robert H. Smith Faculty of Agriculture, Food and Environment, The Hebrew University, Rehovot, Israel.

*. Corresponding Author. daniel.barkan@mail.huji.ac.il +972-8-9489065

§ . Equal contribution by these two authors.

Supplementary Material:

Supplementary data:

Supplementary table 1: oligo-primers used in this work.

Primer name	Seq	Templete	product	remarks
fmtsmgup	5 – CAGCGACGAGTTCGTCGCCGAGCTCACCGA-3	*M. smeg fmt* (Msmeg_3064)	0.6kb	No product on *Mtbfmt*
fmtsmgdo	5 – CCAGCCGCACCGGATGCGACCCGGTGCCGA-3			
fmtdelchq1	5 – GTTCTTCGCGTTCTCCGTGGGATGC-3	gDNA *M. smeg wt* or *Δfmt*	Wt: 3.2kb *Δfmt*: 2.3kb	
fmtdelchq2	5 – GGTTGGCGCCGCCACCGTGACTCGG-3			
fmtTBup	5 – GACGTGATCGCCGTGTTGACCCGTCCGGAT-3	Mtb fmt (Rv1406)	0.75kb	No product on *MSmfmt*
fmtTBdo	5 – TTCTTGCCGGGCGGCTGAATCTGGCCCAGC-3			
fmtKOchqD	5 – CGCCGCGCCCAACGCGTCAGCAA - 3	gDNA BCG wt or *Δfmt*	Wt:2.0kb *Δfmt*(hyg): 2.8kb	
fmtKOchqU	5 – TCCTGGTATTGCAGGGTCAGCC - 3			
Int end	5 – TGCGACGACCAAGGAGCTGAT - 3	Bind to the integrase and oriE of attb-integrating plasmids, including pDB284 and pDB299	Used to confirm exchange of pDB284 (1532bp product)and pDB299 (830bp)	Bind to both plasmids, but produce a product of different sizes.
oriE strat	5 – CTGACGCTCAGTGGAACTAG - 3			
strt	5 – GTGCGCCTTGTCTTTGCCGG – 3	*fmt*	0.94kb	Internal primers of *Mtbfmt*
R1	5 – GTGATGGTGTGTTGCCCGTGCGGCCAG – 3	*fmt*		
mid1	5 – CCGAACTCGGCAGAGTTCGT – 3	*fmt*	0.48kb	
mid2	5 – GGATCCGCCGCTCCACGA – 3	*fmt*		

Supplementary table 2: plasmids used in this work

Plasmid name	replication	Genes/constructs	Resistance	use
pDB117	*Attb* integrating	AHT-Mtb*fmt*	Kanamycin	Conditional FMT expression
pDB178	No mycobacterial replication	*mCherry, galK, sacB*	Zeocin streptomycin	Two-step deletion of *fmt* in *M. smegmatis*
pYUB412	Attb integrating	None	Hygromycin	Removal of pDB117 from genome
pDB245	oriE (no oriM)	Allelic exchange construct for *fmt*	Hygromycin	Creation of phDB30
pDB284	Attb integrating	Mtb*fmt*	Kanamycin	Creation of Mero-diploid strain of BCG
pDB290	Attb integrating	Mtb*fmt*	Zeocin	Exchange of pDB284
pDB299	Attb integrating	*lacZ*	Zeocin	Complete removal of pDB284 to create full KO
pDB332	Episomal, oriM	*fmt,mCherry*	kanamycin	*fmt* complementation in full deletion-mutant

Supplementary table 3: Mutant and strain used in this work:

Name of mutant	genotype	Creation
M. smegmatis MC^2-155	Wild type	Lab strain
M. smegmatis mDB21	*Δfmt*, attb:AHT-Mtb*fmt*:kana	Insertion of pDB117 into wt, deletion of *fmt* using pDB178 two-step
M. smegmatis mDB22	*Δfmt*, attb:hyg	Exchange of pDB117 by pYUB412 in mDB21
BCG subsp. *russia*	Wild type	Lab strain
BCG mDB113	attb:*fmt*:kana	BCG Russia + pDB284
BCG mDB123	*Δfmt*:hyg, attb:*fmt*:kana	Deletion of *fmt* by phDB30, in mDB113
BCG mDB147	*Δfmt*:hyg, attb:*fmt:zeo*	mDB123 + pDB290
BCG mDB150	*Δfmt*:hyg, attb:*lacZ:zeo*	mDB123 + pDB299
BCG mDB168 (mDB150 comp-*fmt*)	*Δfmt*:hyg, attb:*lacZ:zeo, plasmid OriM fmt:kana*	mDB150 + pDB332

Supplementary figures:

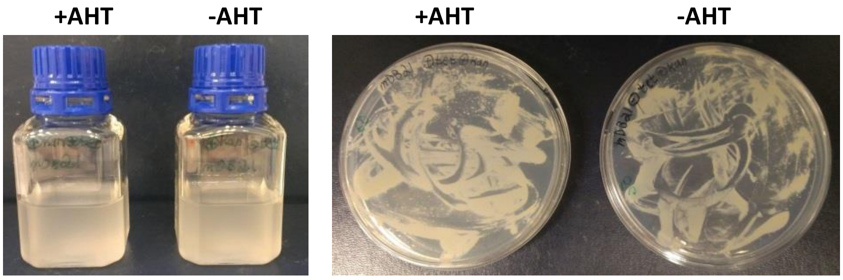

Supplementary figure 1: mDB21 can grow with or without the addition of Anhydeo-tetracycline (AHT).

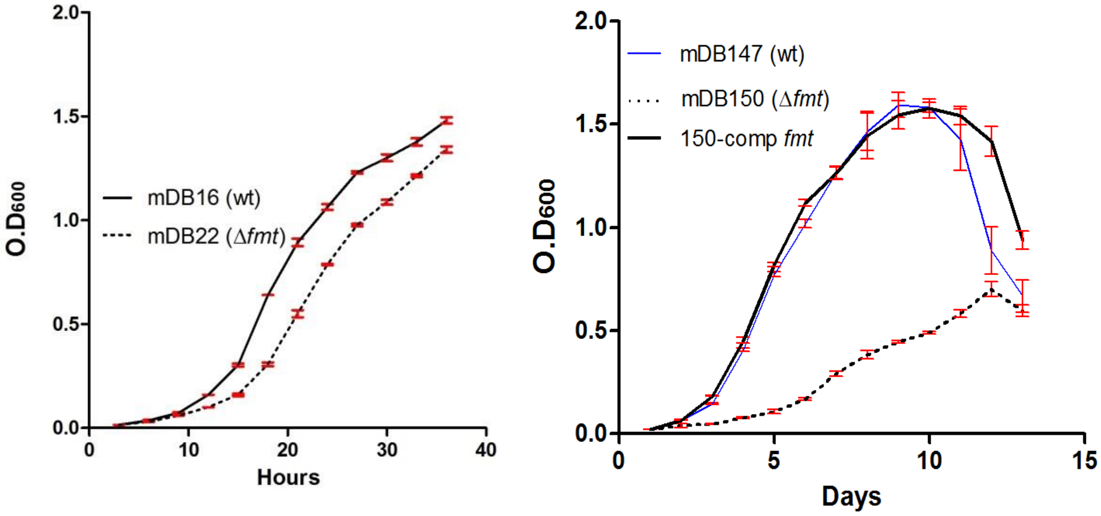

Supplementary figure 2: The linear/linear growth curve of mDB22 vs. mDB16 (right) and mDB150 vs. mDB147 vs. mDB150 comp-*fmt* (left). Error bars are Standard Error of the Mean, SEM.
